# The Significance of Mitochondrial Dysfunction in Cancer

**DOI:** 10.3390/ijms21165598

**Published:** 2020-08-05

**Authors:** Yongde Luo, Jianjia Ma, Weiqin Lu

**Affiliations:** 1 School of Pharmaceutical Science, Wenzhou Medical University, Wenzhou 325000, China; 2 Division of Gastroenterology and Hepatology, Department of Medicine, Stony Brook University, Stony Brook, NY 11794, USA; Jianjia.Ma@stonybrookmedicine.edu

**Keywords:** mitochondria, dysfunction, cancers, TCA cycle, electron transport chain, oxidative phosphorylation, oncogene, tumor suppressor

## Abstract

As an essential organelle in nucleated eukaryotic cells, mitochondria play a central role in energy metabolism, maintenance of redox balance, and regulation of apoptosis. Mitochondrial dysfunction, either due to the TCA cycle enzyme defects, mitochondrial DNA genetic mutations, defective mitochondrial electron transport chain, oxidative stress, or aberrant oncogene and tumor suppressor signaling, has been observed in a wide spectrum of human cancers. In this review, we summarize mitochondrial dysfunction induced by these alterations that promote human cancers.

## 1. Introduction

Mitochondria are semi-autonomous intracellular double membrane-bound organelles, which include an outer membrane, a highly folded inner membrane (crista), a matrix space surrounded by the inner membrane, and an inter-membrane space between the inner and outer membranes [1]. Usually, a cell has hundreds or thousands of mitochondria, which can occupy up to 25% of the cellular cytoplasm. Mitochondria are a convergence point for glucose, glutamine, and lipid metabolism [2]. The primary function of mitochondria is to support the TCA cycle and aerobic respiration by oxidative phosphorylation, generating ATP through the mitochondrial respiratory chain to fulfill the energy needs for cell survival [3,4]. One unique feature of mitochondria is that they possess their own supercoiled, double-stranded circular genetic material called mitochondrial DNA (mtDNA) that encodes rRNAs, tRNAs, and proteins essential for electron transport and oxidative phosphorylation, as well as their own genetic repair mechanisms [3,5,6]. Mitochondrial biogenesis requires the coordinated expression of both mtDNA- and nuclear DNA-encoded genes. Thirteen proteins are encoded by mtDNA, while approximately 1000 mitochondrial proteins are encoded by the nuclear genome, translated in the cytoplasm and transported into the mitochondria by a specific transport system [7]. These two pools of proteins are required to maintain mitochondria as a cellular power hub and a signaling nexus that are essential for normal cell function. Defects in many of the mitochondrial components are causal for a multitude of cellular diseases. Of note, the reprogramming of cellular metabolism and the aberrant redox status have been heralded as major emerging hallmarks of neoplastic transformation. Overall, mitochondrial dysfunction caused by mtDNA mutations, malfunctioned TCA cycle enzymes, electron respiratory chain leakage and subsequent oxidative stress, and/or aberrant oncogenic and tumor suppressor signaling is known to alter cellular metabolic pathways, disrupt redox balance, and cause resistance to apoptosis and therapies that significantly contribute to the development of multiple types of human cancers. In the following sections, we will present current knowledge on these aspects of mitochondrial dysfunction pertaining to the pathologies of various forms of human malignancies.

## 2. Mitochondrial TCA Cycle and Human Cancer

**Figure 1:**
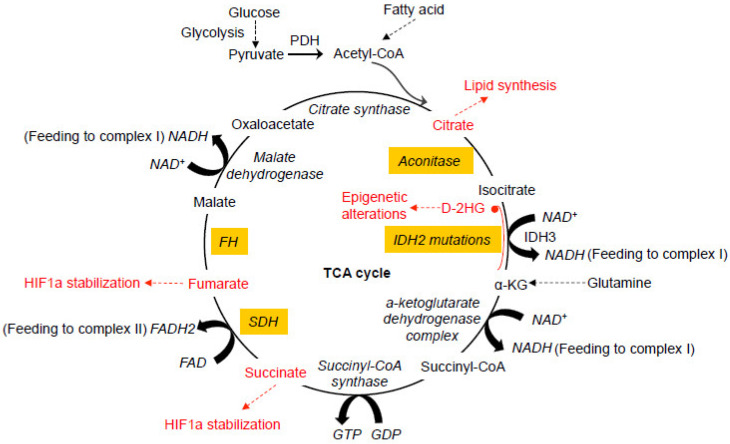
Dysfunctional tricarboxylic acid (TCA) cycle enzymes in cancers. The normal TCA cycle deemed for the breakdown of acetyl-CoA is subject to disruption by oxidative stress and dysfunction of the TCA cycle enzymes, contributing to various cancers. Mutations in IDH2 can lead to the production of D-2HG from α-KG. Abnormal accumulation of 2-HG causes epigenetic landscape alterations and inhibits SDH, resulting in the accumulation of succinyl-CoA and mitochondrial respiration impairment. Mutations in SDH cause abnormal accumulation of succinate and subsequent inhibition of PHDs, resulting in HIF1a stabilization. Like SDH, mutations in FH also cause HIF1a stabilization. Oxidative stress induced by the dysfunction of mitochondrial electron transport chain causes abnormal modification of the iron-sulfur center in aconitase, leading to the exportation of citrate from mitochondria for cholesterol and fatty acid synthesis. All of these abnormal alterations in the TCA cycles have been more or less implicated in neoplastic transformation of different types at different degrees. PDH, pyruvate dehydrogenase. PHD, HIF prolyl hydroxylase. D-2HG, D enantiomer of 2-hydroxyglutarate. α-KG, alpha-ketoglutarate. IDH, isocitrate dehydrogenase. SDH, succinate dehydrogenase. FH, fumarate hydratase.

Glucose represents a key component of the daily diet and can be actively transported into cells and converted into pyruvate by glycolysis in the cytosol. When oxygen is limited, pyruvate is converted into lactate by lactate dehydrogenase (LDH). In the presence of oxygen, pyruvate can be transported into mitochondria and decarboxylated to produce acetyl CoA by the pyruvate dehydrogenase complex (PDH) in the mitochondria. The oxidation of acetyl CoA is achieved by the tricarboxylic acid (TCA) cycle, which involves a set of metabolic reactions catalyzed by specific enzymes including citrate synthase, aconitase, isocitrate dehydrogenase (IDH), α-ketoglutarate (α-KG) dehydrogenase complex, succinyl-CoA synthase, succinate dehydrogenase (SDH), fumarate hydratase (FH), and malate dehydrogenase in the mitochondria to generate CO_2_, H_2_O, and the bioenergetic products GTP, NADH, and FADH2 (Figure 1) [8,9]_._ The TCA cycle represents the final converging route for the oxidation of lipids, carbohydrates, and amino acids [10]. Recent studies have shown that mutations in several enzymes involved in the TCA cycle can induce various human cancers.

### 2.1. Gain-of-Function IDH Mutations

IDHs are a family of enzymes with three isoforms, which catalyze the oxidative decarboxylation of isocitrate to α-KG. These three isoforms are spatially distributed to distinct subcellular locations. IDH1 and IDH2 are NADP^+^-dependent enzymes, mainly participating in reductive glutamine metabolism in the cytosol and the mitochondrial compartment, respectively, while IDH3 is an NAD^+^-dependent core mitochondrial enzyme in the TCA cycle. Genome-wide mutation analyses and high-throughput deep sequencing have identified that 70% of grade II-III gliomas and secondary glioblastomas, as well as 10% of acute myeloid leukemia (AML), have mutations in IDH1 or IDH2 [11,12,13]. The mutations in IDH1 and IDH2 confer a gain-of-function alteration to the enzymes resulting in the production of oncometabolite D-2-hydroxyglutarate (D-2HG) from α-KG [14]. As an α-KG competitor, the elevated D-2HG effectively inhibits dioxygenase enzymes, such as the 5′-methylcytosine hydroxylase TET2, leading to epigenetic landscape alterations through increasing histone and DNA methylation (Table 1) [15,16,17,18,19]. In AML tumors, TET2 and IDH1/2 mutations are mutually exclusive, confirming that inhibition of TET2 is critical for mutant IDH1/2-driven cancer pathogenesis. Accumulation of D-2HG has also been shown to promote protein hypersuccinylation via inhibition of SDH and subsequent accumulation of succinyl-CoA, leading to mitochondrial respiration impairment and apoptosis resistance [20].

As an endogenous enantiomer of D-2HG with nearly identical physical properties, L-2HG is 5-10 fold more potent than D-2HG in inhibiting several α-KG-dependent dioxygenases including the TET2 enzyme. Interestingly, under hypoxic conditions, L-2HG can be selectively induced by the reduction of glutamine-derived α-KG primarily by LDHA via promiscuous substrate usage rather than by IDH1 or IDH2, which is independent of hypoxia-inducible factor (HIF) activation. The accumulated L-2HG was found to regulate repressive histone methylation levels, such as H3K9me3, in response to hypoxia, which helps to mitigate reductive stress through inhibition of electron transport and glycolysis [49,50]. It was thus proposed that hypoxia- and LDHA-induced L-2HG is a metabolic signaling intermediate that modulates the epigenetic marks in the nucleus to convey information about the metabolic state of the cell. Furthermore, L-2HG elevation can also be mediated by the reduced expression of L-2HG dehydrogenase (L2HGDH). Overexpression of L2HGDH in renal carcinoma cells reduced histone methylation and suppressed in vitro tumor phenotypes [51]. As oncometabolite D-2HG, the induction of L-2HG under hypoxic or reduced L2HGDH conditions may contribute to the development of epigenetic heterogeneity and malignancy of different tumor types [50,51]. However, how the L-2HG mediated events contribute to the pathogenesis of various cancers remain largely unclear.

### 2.2. Loss-of-Function SDH Mutations

SDH is a mitochondrial hetero-tetrameric enzyme complex consisting of SDHA, SDHB, SDHC, and SDHD subunits, and participates in both the TCA cycle and the electron transport chain at complex II in the mitochondrial inner membrane [52,53]. It oxidases succinate to fumarate in the TCA cycle and reduces ubiquinone (CoQ) to ubiquinol (CoQH2) in the electron transport chain. Inherited mutations in SDH are found to promote the development of hereditary pheochromocytoma and paraganglioma [21,22,23]. Genetic and sporadic mutations of SDH are also found in several other tumors, such as colorectal cancer [24], renal carcinoma [25], gastrointestinal stromal tumor [26], pituitary tumor [27], and ovarian cancer (Table 1) [28]. Mechanistically, mutations in genes encoding SDH complex reduce its catalytic activity, leading to abnormal accumulation of succinate in mitochondria. The accumulated succinate can be transported to the cytosol via the dicarboxylic acid translocator in the mitochondrial inner membrane and the voltage-dependent anion channel (VDAC) in the mitochondrial outer membrane, leading to inhibition of the enzyme activity of HIF1α prolyl hydroxylases (PHDs) [29,30]. PHD inhibition prevents HIF1α degradation and allows the formation of a stable HIF1α and HIFβ complex, generating a pseudo-hypoxic state that can promote neoplastic transformation via transcriptional activation of genes involved in proliferation, metabolism, angiogenesis, and invasion (Table 1) [31]. Interestingly, with similar structures as succinate, the other two oncometabolites, 2-HG and fumarate, can also function through such a parallel HIF1-inducing oncogenic mechanism [32]. Succinate accumulation was also reported to induce the formation of reactive oxygen species (ROS), which stabilize HIF1α through oxidizing and thus inactivating Fe^2+^-associated PHD. In addition, succinate can serve as a ligand of G protein-coupled receptor 91 (GPR91) to trigger downstream physiological and pathophysiological cascades, such as those involved in inflammation and innate immunity [33]. Like 2-HG, succinate can competitively inhibit 2-oxogluterate-dependent dioxygenases, such as histone lysine demethylases and TET hydroxylases, resulting in epigenetic dysregulation and hypermethylation affecting genes involved in cell growth, differentiation and tumorigenesis [34].

### 2.3. Loss-of-Function FH Mutations

FH forms a homo-tetramer to catalyze the reversible hydration of fumarate to malate in the TCA cycle in the mitochondrial matrix. An isoform of FH is also present in the cytosolic compartment to regulate cytosolic fumarate levels. Patients with the homozygous germline loss of *FH* developed fumaric aciduria, an autosomal recessive metabolic disease associated with mental retardation and speech impairment [54]. Patients with heterozygous germline mutations in the *FH* gene are susceptible to multiple cutaneous and uterine leiomyomatosis (MCUL), which is associated with an increased risk of developing papillary renal cell cancer and leiomyosarcoma (Table 1) [35,36,37]. The sporadic loss of FH or transcriptional downregulation of *FH* was found in various cancers, such as pheochromocytomas, paragangliomas, neuroblastomas, adrenocortical carcinoma, ependymoma, osteosarcoma, bladder cancer, breast cancer, glioma, colorectal cancer, and testicular cancers [38,39,40,41,42,43]. Inactivation of FH compromises the TCA cycle and respiration, and tumor cells harboring mutations in FH were found to adapt to the glutamine-dependent reductive carboxylation reactions as an alternative pathway for the formation of αKG and citrate, which fuel the TCA cycle and generate NADH to feed into the electron transport chain for ATP generation and the maintenance of mitochondrial membrane potential [44]. This glutamine-dependent pathway uses both mitochondrial and cytosolic NADP^+^-dependent IDH1 or IDH2 to metabolize glutamine-derived αKG to citrate that is important for lipid synthesis [45]. Glutamine also provides carbon for heme biosynthesis and degradation pathways to support FH-deficient cell survival [44]. The tumorigenic effect caused by defective HF is, in part, attributed to the abnormal accumulation of fumarate, resulting in the inhibition of PHDs in the cytosol and thus, stabilization and activation of HIF1α [30,46], which plays a major role in cancer metabolism (Table 1).

## 3. Mitochondrial DNA Mutations and Respiratory Chain Dysfunction in Cancer

### 3.1. Mitochondrial Genome

**Figure 2:**
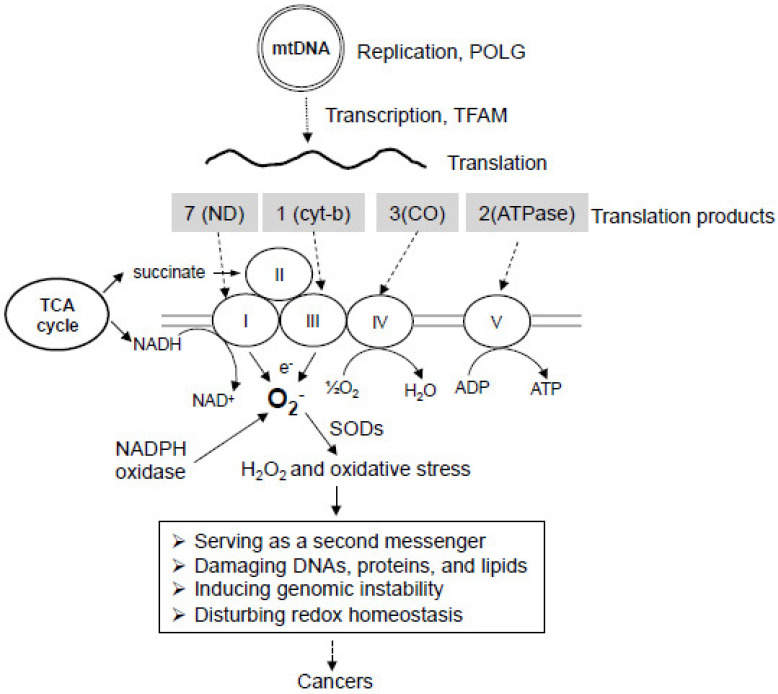
Mitochondrial genome, electron transport chain, and oxidative stress in cancer development. Mitochondria have their own supercoiled, double-stranded genome called mtDNA. Polymerase gamma (POLG) is the only polymerase responsible for mtDNA replication. Mitochondrial transcription factor A (TFAM) is responsible for mtDNA transcription. The products of mtDNA translation are all involved in the assembly of the mitochondrial respiratory chain, including complex I, complex III, complex IV, and complex V, while all components of complex II are encoded by nuclear DNA. NADH as a product of the TCA cycle feeds into complex I, while succinate as a substrate feeds into complex II of the electron transport chain. Electron leakage from complex I and III can be captured by molecule oxygen to generate superoxide (O_2_^−^), which may lead to oxidative stress. Mitochondrial dysfunction can also enhance NADPH oxidase (NOX) activity for the generation of O_2_^−^. SODs can convert O_2_^−^ to H_2_O_2,_ which can be then converted to other forms of ROS. ROS can act as a second messenger to alter normal signaling cascades, or directly damage DNAs, proteins, and lipids, thus inducing genomic instability, redox imbalance, and the development of cancers and drug resistance.

Human mtDNA contains a 16.6 kb circular genome that encodes 37 genes (13 respiratory enzyme complex proteins, 22 tRNAs, and 2 rRNAs) essential for the production of proteins involved in electron transport and oxidative phosphorylation (Figure 2) [3,55,56]. All the mtDNA encoded proteins are involved in the assembly of the mitochondrial respiratory chain, including NADH dehydrogenase (ND) subunits ND1, ND2, ND3, ND4, ND4L, ND5, and ND6 of complex I, cytochrome b of complex III, cytochrome c oxidase subunits I, II and III (COI, COII, and COIII respectively) of complex IV, and ATPase 6 and ATPase 8 of complex V [57,58]. The mtDNA sequence is extremely economical in that the genes have none or only a few non-coding bases between them. The non-coding region, so-called displacement loop (D-loop), contains the origin of heavy strand replication, the heavy strand promoter (HSP), the light strand promoter (LSP), and the regulatory sequences necessary for mtDNA replication and transcription [59].

DNA polymerase gamma (POLG) is a nuclear-encoded enzyme that translocates to mitochondria to serve as the only DNA polymerase, which directs mtDNA replication and repair [60,61,62]. POLG is a heterodimeric enzyme containing a catalytic core and an accessory subunit. The multi-functional catalytic subunit has an exonuclease domain, a linker region, and a polymerase domain, and exhibits DNA polymerase activity, 3′-5′ proofreading exonuclease activity, and apurinic/apyrimidinic (AP) lyase activity [63,64]. The accessory subunit is required for DNA synthesis by increasing the affinity of POLG to mtDNA [65]. mtDNA replication starts at the D-loop, and its initiation requires an RNA primer produced by mtDNA transcription [66,67].

The process of transcription initiation in mitochondria requires the mitochondrial RNA polymerase, mitochondrial transcription factor A (TFAM), and mitochondrial transcription factor B1 or B2 [68,69]. They form a complex at the mitochondrial promoter D-loop region to initiate transcription. TFAM is a nuclear-encoded protein translocated to mitochondria and is essential for both mitochondrial DNA transcription and replication [70]. It contains two DNA-binding, high-mobility group (HMG) box domains. Like other HMG family proteins, TFAM can bind, unwind, and bend mtDNA without sequence specificity [71]. However, TFAM shows a higher affinity for both LSP and HSP, thus preferentially bending and unwinding mitochondrial transcriptional promoters in the D-loop region to enhance mtDNA transcription [72,73,74]. TFAM is also involved in nucleoid formation by packaging mtDNA into protein-DNA aggregates, thus serving as mitochondrial histone [75,76]. The dominant-negative form of POLG was found to compromise the normal function of POLG and induce TFAM degradation, mitochondrial respiration dysfunction, aerobic glycolysis elevation, redox alteration, and NADPH oxidase (NOX) activation [77,78].

### 3.2. Mitochondrial DNA Mutations in Cancer

Mutations in mtDNA have been found in a spectrum of human cancers [3,79,80,81]. They can occur at the D-loop region, the protein-coding region, the rRNA genes, and the tRNA genes [82,83,84]. The most common mtDNA alterations in cancers are point mutations and reduced copy numbers, but frame-shifting insertions and large-scale deletions have also been identified [82]. The D-loop region of mtDNA is regarded as the most frequent site of somatic mutations in many cancers. Because mtDNA replication starts at the D-loop, mutations in the D-loop region can affect mtDNA copy number in cancers. In a study investigating mtDNA mutations in 31 gastric cancer patients, 51.6% of mtDNA mutations occurred in the D-loop region, 22.6% in the protein-coding region, and 4% in tRNA genes [85]. Through sequencing analysis of the entire mtDNA from 58 breast cancer samples with paired non-tumorous breast tissues, researchers identified that 52.5% of mtDNA mutations were located in the D-loop region, 37.5% in protein-coding region, and 5% each in the rRNA and tRNA genes. These somatic mutations are thought to cause mitochondrial dysfunction by altering the functions of 12S rRNA (T1499C), 16S rRNA (G1913A), tRNA^Trp^ (G5522A), tRNA^Cys^ (G5809A), as well as ND2 (G5112A), ND4L (G10599A), ND5 (A13878G) of complex I, cytochrome b (T15416C) of complex III, and COI (G6384A, G6768A) and COIII (G9412A, G9774A, A9901C) of complex IV [84]. In hepatocellular carcinoma, somatic mtDNA mutations that affect the functions of tRNA^Val^ (T1659C), tRNA^Ala^ (G5650A), ND1 (G3842A), ND4 (11032delA, A11708G), ND5 (12418insA), COI (T6787C), COII (G7976A), and COIII (A9263G, G9267A) were also observed [83]. By sequencing the mitochondrial genomes of 384 prostate cancer patients, researchers found that 15.4% of the tumors harbor mutations in the non-coding control region of mitochondrial genome [86], while ND5 was the most frequently mutated gene in the protein-coding region [86]. Furthermore, sequence analyses of 226 paired tumor and normal tissue samples in five cancers by The Cancer Genome Atlas (TCGA) revealed that the frequencies of deleterious tumor-specific somatic mtDNA mutations are 63% in rectal adenocarcinomas, 53% in colon adenocarcinomas, 36% on ovarian serous cyst adenocarcinomas, 30% in acute myeloid leukemia, and 13% in glioblastoma, highlighting the prevalence of mtDNA mutations in diverse types of cancers. These mtDNA mutations are predicted to impact the functions of encoded proteins that confer a selective advantage in oncogenesis [87]. Some mutation hotspots have been observed in several cancers. The 12418insA mutation, with an adenosine nucleotide insertion in a poly-A sequence at nucleotide position 12,418–12,425 of mtDNA, causes a frame-shift and premature termination of the ND5 gene and results in a truncated polypeptide. This mutation was observed in colorectal cancer, breast cancer, gastric cancer, and hepatocellular cancer and is correlated with defective mitochondrial respiration, higher levels of lactate production, and increased tumorigenesis [83,84,85,88]. By using a cybrid cell model, a heteroplasmic 12418insA mutation was shown to increase ROS generation and decrease oxidative phosphorylation in human cancer cells, as well as promote tumor growth in nude mice [89], suggesting that this hotspot mutation plays a critical role in tumorigenesis. Of note, the G-to-A and T-to-C transitions, which are typical changes that occur upon exposure to free radicals, are rather common, suggesting that these mutations are associated with oxidative stress. In a mtDNA analysis of colon cancer patients, it was found that 70% of the identified mtDNA mutations were T-to-C and G-to-A replacements [90]. Similarly, the G-to-A replacements in the ATP6 gene at positions 8557, 8697, and 8854 of mtDNA were found in breast cancer patients [91]. The accumulation of mtDNA mutations was found to correlate with the degree of malignancy [92]. Furthermore, mutations in mtDNA exacerbate ROS production and oxidative stress [93] to promote more mtDNA mutations, which, in turn, create a vicious cycle critical for tumorigenesis.

### 3.3. Mitochondrial Oxidative Phosphorylation Defects in Cancer

**Figure 3:**
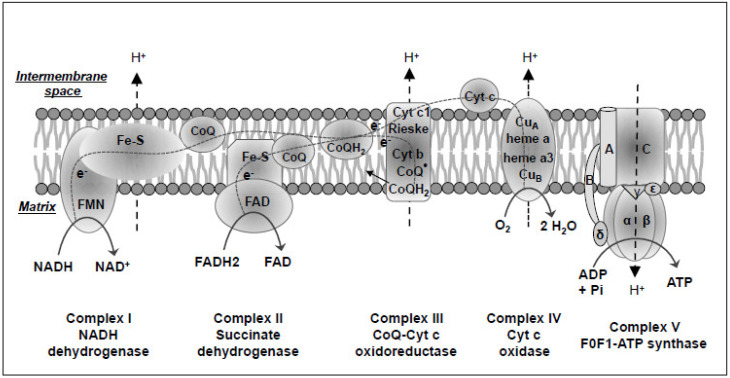
Schematic of mitochondrial electron transport chain and oxidative phosphorylation system. In mammalian cell mitochondria, the oxidative phosphorylation system (OXPHOS) is located largely across the inner membrane and on the side of the matrix and is organized into five multi-enzyme complexes, including complex I (also called NADH dehydrogenase), complex II (Succinate dehydrogenase), complex III (cytochrome bc1 complex or CoQ-Cyt c oxidoreductase), complex IV or cyt c oxidase (CO), and complex V (FoF1-ATP synthase) for oxidative phosphorylation and energy transfer to ATP. The high-energy electron carriers NADH and FADH2 produced by the intermediate substrate metabolism in the TCA cycle in the matrix are sequentially oxidized by Complex I and II to generate an electrochemical proton gradient across the inner membrane, which is eventually used as a driving force by the Complex V to produce ATP. FMN, flavin mononucleotide. Fe-S, iron-sulfur cluster at enzyme center. e-, a high-energy electron of negative charge. CoQ, ubiquinone or coenzyme Q. CoQH2, reduced coenzyme Q or ubiquinol. Cyt, cytochrome. CuA, cupper-containing metallic center A of complex IV. Heme a, heme-containing cytochrome a. A, B, C, α, β, γ, δ, and ε, different protein subunits of complex V. Pi, inorganic phosphate ion.

Embedded in the inner mitochondrial membrane, the mitochondrial oxidative phosphorylation system (OXPHOS) is composed of five multi-enzyme complexes, including complex I (also called NADH dehydrogenase), complex II (SDH), complex III (cytochrome bc1 complex or coenzyme Q-cytochrome c oxidoreductase), complex IV or cytochrome c oxidase (CO), and complex V (FoF1-ATP synthase) [56]. The electrons carried in NADH and FADH2 are used as fuel and donated to the molecular oxygen through the respiratory chain complexes I to IV, generating a proton gradient. The dissipation of the established proton gradient through complex V generates ATP (Figure 3) [94,95]. Thus, mitochondria, as the powerhouse of the cell, play a central role in cellular oxidative metabolism.

Complex I is the largest and the most complicated component of the respiratory chain with 45 different subunits and exhibits a characteristic L-shaped structure [96,97]. It is composed of flavin mononucleotide (FMN), iron-sulfur (Fe-S) clusters, and CoQ, a lipid-soluble electron carrier embedded in the lipid bilayer of the inner mitochondrial membrane (Figure 3) [98,99,100]. This holoenzyme oxidizes NADH produced in the TCA cycle to NAD+ and donates the released electrons to FMN, which is thereby reduced to FMNH_2_. The electrons then move into a series of Fe-S clusters and ultimately into CoQ. The CoQ uptakes two protons from the matrix and is reduced to CoQH2. As the electrons move through the series of Fe-S clusters, four protons are pumped from the mitochondrial matrix to the intermembrane space [97]. Complex I deficiency is the most frequent cause of oxidative phosphorylation disorders with a large diversity of clinical symptoms [101]. Currently, however, there is no available stable genetically engineered mouse model that harbors a specific mutation in any of the seven mtDNA-encoded complex I subunits. One major hurdle in developing mouse models of mtDNA mutations is the inability to manipulate the mtDNA genome. To test if mitochondrial dysfunction induced by mtDNA mutations in complex I is necessary for cancer development, a recent study compared the mitochondrial defective L929dt cells that have mtDNA mutations at the *ND2* subunit of complex I to their parental mitochondrial-intact mouse fibroblast L929 cells [102]. L292dt cells showed defects in mitochondrial supercomplex assembly, a lower capacity to generate energy through OXPHOS, and a lower respiratory capacity with a glycolytic shift. In addition, L929dt cells showed higher in vivo tumorigenic and metastatic potentials than their parental L929 cells. Cybrids with L929dt defective mitochondria in L929 nuclear background reproduced all L929dt properties [102], demonstrating that mutations in the ND2 subunit of mitochondrial complex I are responsible for the aggressive tumorigenic and metastatic phenotype and emphasizing the critical role of mutant mtDNA-induced mitochondrial dysfunction in cancer development.

Complex II, the TCA cycle enzyme SDH, is composed of a bound FAD and three Fe-S clusters and catalyzes the oxidation of succinate to fumarate and the reduction of FAD to FADH2 (Figure 3). The FADH2 is then oxidized back into FAD while giving off two electrons to a series of Fe-S clusters and eventually to CoQ. Although these reactions do not directly contribute to the generation of the proton motive force for ATP formation, they transfer two electrons derived from the reactions to membrane-bound CoQ. CoQ is then reduced to CoQH2, which fuels complex III and complex IV [103]. Of note, all subunits of complex II are encoded by the nuclear genome. As aforementioned, the loss-of-function SDH mutations induce various types of cancers.

Complex III is a cytochrome c reductase or coenzyme Q-cytochrome c reductase. It is composed of cytochrome c1 (which contains one heme group), cytochrome b (which contains two different heme groups), a Fe-S Rieske protein, and two CoQ binding sites (Figure 3) [104]. Excluding cytochrome b, which is encoded by mtDNA, all other subunits are encoded by the nuclear genome. Complex III accepts two electrons from a CoQH2 molecule generated from complex I and/or complex II and then transfers one electron sequentially onto Fe-S Rieske protein, the heme group of cytochrome c1, and cytochrome c, which finally diffuses away and travels to complex IV. The second electron moves to the heme group of cytochrome b and then to CoQ, which is partially reduced to a semiquinone radical (CoQ·). When a second CoQH2 attaches to complex III, it transfers the second pair of electrons through the same process as described above, the only difference being that CoQ· generated from the first round accepts another electron to generate CoQH2. During the whole process, two CoQH2 are oxidized to two CoQ, 4 protons are pumped from the mitochondrial matrix to the intermembrane space, two cytochrome c are reduced, and one CoQ is reduced to CoQH2 [105]. Cytochrome b mutations were reported in different cancers, such as pancreatic cancer and bladder cancer [106,107]. In a murine xenograft and a human cell model of bladder cancer, overexpression of a 21-bp deletion mutation of cytochrome b led to increased levels of ROS accompanied by increased oxygen consumption and lactate production, as well as significant tumor growth. This study provides evidence for the role of a bona fide mitochondrial gene mutation in cancer.

Complex IV is the last electron acceptor of the respiratory chain and is composed of heme-containing cytochrome a and cytochrome a3, as well as two metallic centers (CuA and CuB) (Figure 3) [108]. When the two reduced cytochrome c proteins from complex III diffuse and bind to complex IV, two electrons are donated to CuA center and then transferred to cytochrome a. Eventually, one of the electrons is used to reduce cytochrome a3, and the other is used to reduce CuB. Once cytochrome a3 and CuB are in their reduced form, an O_2_ molecule can bind to the two electrons to form a peroxide bridge between cytochrome a3 and CuB. When two more reduced cytochrome c are oxidized, they transfer two more electrons, which, together with two protons from the matrix, are used to break the peroxide bridge to form CuB-OH and cytochrome a3-OH. When two more protons are obtained from the matrix, they are picked up by CuB-OH and cytochrome a3-OH to form two H_2_O with the regeneration of cytochrome a3 and CuB. Overall, in this complex process mediated by complex IV, four reduced cytochrome c are oxidized, one oxygen molecule and four protons in the matrix are used to generate two H_2_O, and four protons are transferred from the mitochondrial matrix to the intermembrane space. Among all subunits of this multimeric complex, three catalytic subunits (COI, COII, and COIII) are encoded by mtDNA, while the remaining 10 subunits are encoded by nuclear DNA and are involved in the regulation of oxygen consumption and proton translocation [109]. Mutations or altered expression of these subunits either increase or decrease complex IV activity as observed in different types of cancers, such as colorectal cancer, glioblastoma multiforme, and esophageal cancer [110,111,112]. Prostate cancer is associated with both inherited and somatic mutations in the COI gene. Studies using the cybrid method showed that COI mutation decreased the activity of the respiratory complex and increased mitochondrial ROS and nitric oxide generation. Cells harboring the mutation proliferated faster in vitro and caused increased tumor growth in vivo [113]. These data suggest that mtDNA mutation-induced complex IV dysfunction contributes to tumor growth.

Complex V is composed of a proton channel-containing F0 domain located in the inner mitochondrial membrane and a catalytic F1 domain, which connects to F0 and is located on the side of the mitochondrial matrix (Figure 3) [114]. The F1 domain is made of five types of polypeptide chains called α, β, γ, ε, and δ chains. Three α and three β chains combine to form an α3β3 hexameric ring structure, which can bind the ADP and Pi to catalyze ATP synthesis in the matrix. The γ and ε chains form the central stalk connecting the hexameric ring to the F0 domain and stimulating the synthesis and release of ATP. The δ chain holds the hexameric ring from rotating. The F0 domain is comprised of a single A subunit, two B subunits, and 10-14 C subunits. The C subunits organize into a ring structure and serve as a proton channel that allows protons to move from the intermembrane space to the matrix. The single A subunit binds to the outside of the C ring structure and helps to connect the F0 domain and F1 domain. The two B subunits, by binding to the A subunit of the F0 domain and the δ subunit of the F1 domain, make up a stalk connecting the F0 domain and the F1 domain. The moving of protons at the intermembrane space through the C ring of the F0 domain stimulates the synthesis of ATP at the hexameric ring of the F1 domain and promotes ATP release to the matrix. Thus, complex V utilizes the energy provided by the proton electrochemical gradient to phosphorylate ADP to ATP, the main energy source for intracellular metabolic pathways. Of note, only two of the F0 subunits are encoded by the mtDNA ATP6 and ATP8 genes [55]. ATP6 and ATP8 gene mutations have been observed in various cancer types, such as prostate cancer osteosarcoma and breast cancer [91,115]. To test if mtDNA mutations that affect complex V functions confer cancer cells a growth advantage, investigators introduced the pathogenic ATP6 T8993G mutation into the PC3 prostate cancer cell line through cybrid transfer and tested the effects on tumor growth in nude mice. The resulting T8993G mutant cybrids were found to generate tumors that were seven times larger than the wild-type (T8993T) cybrids, which barely grew in the mice [116]. The mutant tumors also generated significantly more ROS with impaired mitochondrial ATP synthesis. This study suggests that mutations of mtDNA encoding complex V subunits have the potential to increase tumorigenicity in prostate cancer.

### 3.4. The Complexity in the Roles of mtDNA Mutations in Tumorigenesis

Although the majority of studies suggested a positive role of mtDNA mutations in neoplastic progression, it should be noted that a clear link between mtDNA mutations and tumorigenesis has yet to be established, in part owing to the hurdle in our ability to directly manipulate mtDNA and the complexity of mutational landscape obtained from different experimental models and tumor samples. It thus remains a gap in knowledge on mtDNA mutations and their biological significance in tumor formation, progression, and metastasis. In some preclinical cellular and mouse xenograft models, depletion of mtDNA in tumor cells was shown to decrease tumorigenic phenotypes or potential [117], while the acquisition of normal mtDNA, e.g., via horizontal transfer of the whole mtDNA from surrounding cells, could restore functional mitochondrial respiration and tumorigenic potential of mtDNA-depleted cancer cells [118,119,120]. These studies indicate that mitochondrial respiration or oxidative phosphorylation may be required for tumorigenic potential under certain conditions. Some mtDNA mutations can be found in both normal and cancer tissues. The deletion of mtDNA at 4977-bp has been implicated in the contribution to malignant transformation in breast cancer patients; however, it was also observed in the benign tissues and surrounding normal tissues [121]. Furthermore, analysis of random point mutations in the mtDNA of cancerous and healthy tissues from 21 colorectal cancer patients revealed that the frequency of mtDNA mutations decreased in colorectal tumors relative to the adjacent healthy tissues [122], suggesting a mutation selection during tumor development and that not all mtDNA mutations contribute to cancer development—some may suppress tumor growth [122]. Using different transmitochondrial cytoplasmic cybrid cell models harboring a panel of pathogenic mtDNA mutations that disrupt the respiratory chain, studies showed that defects in the respiratory chain could either promote or inhibit cell death, depending on the specific alterations in the electron flow and cytosolic cues (e.g., ER stress) [123].

Oncocytoma is a rare, predominantly benign neoplasm characterized by the dramatic accumulation of defective mitochondria that have mtDNA mutations in the control region and protein-encoding region that induce respiration chain defects [124]. The common mtDNA gene mutations in oncocytomas include *COI, COII, COIII, ND4, ND5,* and *CYTB*. It has been a puzzle about what restricts oncocytomas to remain a benign disease despite such a spectrum of mutations. A recent study suggests that these genetic defects in mitochondrial respiration block Golgi to lysosome trafficking and autophagy, and activate AMPK and p53, thus limiting tumor growth to only a benign state [125]. The type 2 oncocytomas are closely related to the eosinophilic subtype of chromophobe renal cell carcinoma (ChRCC), as they share similarities in the mutational landscape and transcriptome profile, with ChRCC having acquired additional driver mutations in p53 and PTEN and further genetic instability [125]. This study indicates that the impaired mitochondrial function may be a barrier to tumorigenesis by activating p53 in oncocytomas, while subsequent alterations of p53 and other nuclear genes lead to malignant ChRCC.

In brief, although the majority of evidence supports a role of mtDNA mutations in tumorigenesis and malignant progression, the battle to determine whether an mtDNA mutation is a driver mutation for cancer initiation and progression or merely a passenger mutation that does not determine the development of cancer is predicted to continue. The biological impact of a given mtDNA mutation may vary, depending on the nature of the mutation, the proportion of the mutation in the cell, the effects of the mutation to the respiratory chain, and the interplay of the mutant mtDNA-directed events with cytoplasmic signal pathways. Innovation in mtDNA manipulation in well-defined model systems is essential for clarifying the complexity.

## 4. Mitochondrial Oxidative Stress and Cancer

The process of mitochondrial oxidative phosphorylation consumes more than 90% of oxygen. Although the most portion of oxygen is fully reduced to water by cytochrome c oxidase, 1–2% is incompletely reduced to superoxide radicals mainly in complex I and III of the respiratory chain [126,127,128,129]. Superoxide in the mitochondrial matrix can be rapidly dismuted by mitochondrial superoxide dismutase 2 (SOD2) yielding H_2_O_2_, which can be released into the intermembrane space and cytosol leading to the generation of cytoplasmic ROS (Figure 2). In the presence of a reduced transition metal, H_2_O_2_ can be converted into the highly reactive hydroxyl radical OH· [130,131,132,133]. The unpaired electrons are highly reactive, producing chemical modifications that damage proteins, lipids, and nucleotides [134]. Under physiological conditions, free radicals and their derivatives exist in living tissues at low but measurable concentrations, which are determined by the balance between the rate of radical production and the rate of clearance. Free radicals can be eliminated by the reducing equivalent GSH, an abundant cellular thiol and a major determinant of cellular redox equilibrium [135]. Other antioxidant enzymes such as glutathione peroxidase (GPX), glutathione reductase (GR), peroxiredoxin (PRX), thioredoxin, and thioredoxin reductase also play important roles in the clearance of free radicals and the maintenance of redox homeostasis [136].

Mitochondrial respiratory chain dysfunction induced by mtDNA mutations can cause an increase in electron leakage, resulting in an elevation of free radical generation as widely observed in cancer cells [93,137]. In most cells, mitochondrial complex I and complex III, as well as membrane-bound NADPH oxidases (NOXs) are the main sources of cellular ROS [104,108,138,139,140,141]. Interestingly, mitochondrial respiratory chain dysfunction can induce NOX activation [77,142], suggesting that NOX can be activated in response to mitochondrial dysfunction, contributing to heightened levels of cellular ROS. The escalated ROS generation in cancer cells serves as a messenger to stimulate cell proliferation and as an endogenous source of DNA-damaging agents to promote genetic instability and the development of cancer (Figure 2) [143]. DNA contains a large number of ROS-reactive sites [144]. Oxidative damages to DNA may be in the form of base modifications (such as 8-oxoguanine), abasic sites, strand breaks, or various other types of lesions [145]. Of note, ROS production in mitochondria renders mtDNA more susceptible to damage and mutagenesis than the nuclear genome. This is because mtDNA lacks histone protection, has limited DNA damage repair capacity, and is in close proximity to the electron transport chain. As mitochondrial transcription is polycistronic, deletion or insertion of a nucleotide may readily cause the downstream codon frameshifting, resulting in an inability to encode functional products [146]. It was reported that the rate of mtDNA mutation is 10–20 times greater than that of nuclear DNA [147]. Thus, ROS-induced mtDNA mutations and the high susceptibility of mtDNA to mutations play key roles in many diseases, including cancer, aging, and neurodegenerative diseases.

Notably, some key enzymes for energy metabolism can be directly targeted by free radicals. The Fe-S clusters are essential components of the redox-active enzymes within both the TCA cycle and the respiratory chain [148]. Excess superoxide and hydroxyl radicals are capable of reacting with the Fe-S clusters in NADH dehydrogenase, SDH, aconitase, and other enzymes, resulting in their inactivation and subsequent inhibition of production of biological intermediates and energy [149]. NADH dehydrogenase is one of the key enzymes involved in the oxidative phosphorylation, and aconitase is the first rate-limiting enzyme converting citrate into isocitrate in the TCA cycle generating high energy reducing equivalent NADH. Aconitase has been found to be significantly reduced in tumor cells [150]. The impaired activity of aconitase causes a truncation to the TCA cycle, leading to the exportation of considerably large amounts of citrate [151], an essential precursor for the de novo biosynthesis of cholesterol and fatty acid.

SOD1 is a major superoxide-scavenging enzyme catalyzing the conversion of O^2−^ to H_2_O_2_ in multiple cellular locations, including the cytoplasm, mitochondrial intermembrane space, nucleus, and lysosomes. More than 30% of mice deficient in SOD1 developed liver tumors by 20 months of age [47]. Extensive oxidative damage, including increased DNA damage, protein oxidation, and lipid peroxidation, and decreased cytosolic aconitase activity, were observed in the derived liver cancer cells. Abnormal mitochondria with disorganized cristae and reduced size were also seen in these cancer cells (Table 1). As a family member of SOD1, SOD2 catalyzes the dismutation of superoxide in the mitochondrial matrix. Heterozygous *SOD^+/−^* mice exhibited mitochondrial oxidative damage and decreased mitochondrial membrane potential, along with significant elevation of 8-oxo-deoxyguanosine in both nuclear DNA and mtDNA [48]. Tumor incidence, in particular of lymphoma and pituitary adenoma, increased 100% in the old SOD2^+/−^ mice compared with the wild-type mice (Table 1). These studies again suggest that defects in ROS-scavenging enzymes predispose cells to oncogenic transformation.

## 5. Oncogene and Tumor Suppressor in Regulating Mitochondrial Function

Oncogenes and tumor suppressors have been found to either directly or indirectly regulate mitochondrial function and contribute to mitochondrial dysfunction. Here, we highlight several examples to provide another layer of knowledge on mitochondrial regulation.

### 5.1. RAS and Mitochondrial Function

The RAS family of small GTPases, including HRAS, NRAS, and KRAS, functions as a binary molecular switch of multiple downstream signaling pathways. When turned on by GTP occupancy, RAS activates multiple downstream signaling cascades, such as the MAPK and PI3K/AKT/mTOR pathways, to control a wide range of cellular responses, including proliferation, survival, migration, and metabolism [152,153]. RAS family members are among the most frequently mutated genes in human cancers, such as pancreatic, lung, and colorectal cancers. Mutations of KRAS have been found in over 95% of pancreatic ductal adenocarcinomas and are considered as a potential biomarker for patients with pancreatic cancer. However, thus far, mutant KRAS has not proven to be a useful biomarker because the mutation can be also frequently detected in healthy individuals [154,155]. The prevalence of mutant *KRAS* in normal individuals suggests that mutant KRAS per se is not sufficient for full-blown cancer development. Studies using genetically engineered mouse models have shown that the adult pancreatic acinar cells are refractory to transformation by one allelic KRAS mutation [156,157], suggesting that one allelic mutant KRAS expression is insufficient to drive PDAC in adults. Recent studies have found that mutant KRAS allelic imbalance is common in human cancers [158,159,160], highlighting that the increased mutant KRAS dosage, rather than a single allelic mutant *KRAS* alone, plays a more important role in the accentuated KRAS signaling, tumorigenesis, and metastasis [161]. Oncogenic transformation induced by mutant KRAS overexpression attenuated normal mitochondrial function and increased oxidative stress [77,162]. Cells expressing oncogenic KRAS also exhibited enhanced glycolytic activity, decreased oxidative flux through the TCA cycle, increased utilization of glutamine to favor the production of NADPH and cellular redox adaptation, and defective mitochondrial respiratory chain [162,163,164,165]. Thus, a better understanding of the metabolic vulnerability caused by mutant RAS may provide novel therapeutic strategies to treat many lethal human cancers.

### 5.2. HIF and Mitochondrial Function

Human tumors can endure conditions of profound hypoxia, which indicates that adaptation to hypoxic conditions is a crucial step in tumorigenesis [166]. During hypoxia, when mitochondria respiration is limited, a critical adaptive regulator that is often linked to tumor malignancy is HIF1 [167]. The half-life and transcriptional activity of HIF1α are negatively regulated by O_2_-dependent prolyl and asparaginyl hydroxylation, respectively [168]. PHD is an enzyme that can modify HIF1α at proline sites, which mark HIF1α for pVHL mediated degradation by 26S proteasome [169,170]. During the hydroxylation reaction, Fe (II) in the active site of the hydroxylases is oxidized to Fe (III). The hydroxylases utilize ascorbate as a cofactor to reduce Fe (III) back to Fe (II). H_2_O_2_ has been shown to oxidatively modify this process to inactivate PHD, thus stabilizing HIF1α in the presence of oxygen. Administration of a free radical scavenger AEOL-10113 could block the induction of HIF1 activity [171]. As aforementioned, metabolic enzyme mutations, such as SDH and HF mutations, can also induce HIF1α stabilization in favor of tumorigenesis. The stabilized HIF1α can be transported into the nucleus and forms a heterodimer with HIF1β, which then recruits p300/CBP to form an active complex that binds to the hypoxia-responsive element (HRE) in the promoter region of genes encoding glycolytic enzymes, including aldolase A, enolase 1, lactate dehydrogenase A (LDHA), phosphofructokinase, phosphoglycerate kinase, pyruvate kinase, and glucose transporters [166], promoting glucose breakdown to provide building blocks necessary for cancer cell proliferation. In addition, the stabilized HIF1α can suppress both the TCA cycle and aerobic respiration by inducing pyruvate dehydrogenase kinase 1 (PDK1). The increased expression of PDK1 phosphorylates and inactivates PDH, providing a route by which HIF1 activation prevents pyruvate from entering into the TCA cycle and synthesizing citrate for the production of cytoplasmic acetyl CoA, a major source of acetyl groups for protein acetylation and lipid synthesis [172]. Under this mitochondrial inhibition condition, glutamine-dependent reductive carboxylation by IDH1 and IDH2 was found to be the major pathway to generate citrate for lipid synthesis to support cell growth and viability [173,174].

### 5.3. c-Myc and Mitochondrial Function

c-Myc is an oncogenic basic helix–loop–helix leucine zipper transcription factor. Through dimerization with its partner Max, c-Myc binds to specific DNA sequences (E box) and stimulates transcription of specific genes. A common feature of some human malignancies is the deregulated expression of the c-Myc oncogene [175], which enhances aerobic glycolysis by directly upregulating the expression of glycolytic genes such as enolase A, hexokinase II, lactate dehydrogenase A, phosphofructokinase, and glucose transport I independent of hypoxia [176]. Genes for mitochondrial biogenesis and function, such as cytochrome c and TFAM, constitute another group upregulated in response to c-Myc overexpression [177]. On the other hand, mitochondrial dysfunction induces oxidative stress and H_2_O_2_ production, which enhances IκB degradation, allowing NF-κB translocation to the nucleus to direct c-Myc gene expression, contributing to tumorigenesis [178].

### 5.4. p53 and Mitochondrial Function

p53 protein is encoded by the *TP53* gene and is a well-known tumor suppressor. Wild-type p53 is normally short-lived with a low level of expression, which is mainly due to the negative regulation by murine double minutes 2 (MDM2), an E3 ubiquitin ligase that directs proteasome-mediated p53 degradation. However, under stressed conditions, p53 can be stabilized by post-translational modifications and act as a transcription factor to promote cell cycle arrest, DNA damage repair, and apoptosis. Of note, wild-type p53 was also reported to inhibit glucose uptake and glycolysis through transcriptional inhibition of glucose transporter GLUT1 and GLUT4, activation of the glycolytic inhibitory protein named TIGAR (TP53-induced glycolysis and apoptosis regulator) [179], and reduction of glucose-6-phosphate dehydrogenase (G6PD), an important enzyme in the initiation of the pentose phosphate pathway [180]. Furthermore, wild-type p53 can also stimulate oxidative phosphorylation by inducing the expression of its target genes, such as the synthesis of cytochrome c oxidase 2 (SCO2) [181]. SCO2 is a nuclear gene that encodes a copper-binding protein required for the assembly of mitochondrial DNA-encoded CO II subunit into complex IV, the major site of oxygen utilization and oxidative phosphorylation in eukaryotic cells. Wild-type p53 also stimulates fatty acid β-oxidation in mitochondria and inhibits fatty acid biosynthesis through regulating the expression of genes, such as FASN (fatty acid synthase) and ACLY (ATP citrate lyase), to prevent lipid membrane formation and cell proliferation [182]. *TP53* somatic mutations, especially missense mutations at the DNA-binding domain, are observed in more than 50% of human malignancies [183,184], highlighting the pivotal role of altered forms of p53 protein in tumorigenesis. Overall, *TP53* mutations may induce the stabilization of p53 and subsequent aberrant transcription of its target genes, leading to heightened aerobic glycolysis, aberrant lactate buildup, oxidative stress, and dysregulated oxidative phosphorylation to facilitate cancer cells growth [185].

### 5.5. NRF2 and Mitochondrial Function

Nuclear factor erythroid 2-related factor (NRF2), a cap ‘n’ collar basic leucine zipper (CNC-bZIP) transcription factor, is a master regulator of the cellular antioxidant response. Under non-stressed conditions, NRF2 protein levels are kept low due to ubiquitination and proteasomal degradation. Three ubiquitin ligase complexes, including KEAP1-CUL3-RBX1, β-TrCP-SKP1-CUL1-RBX1, and HRD1, are responsible for NRF2 degradation [186]. NRF2, by forming a heterodimer with MAF proteins, controls the expression of more than 200 genes that contain the so-called antioxidant response elements (AREs) in their regulatory regions. By regulating the expression of γ-glutamyl cysteine ligase catalytic (GCLC) and modulatory (GCLM) subunits, glutathione reductase (GR), cystine-glutamate antiporter xCT, and glutathione synthetase (GS), NRF2 enhances the synthesis of glutathione, which detoxifies ROS to attenuate the oxidative damage. In cancers, expression of KRAS^G12D^, BRAF^V619E^, or MYC can lead to a heightened NRF2 expression in association with a higher level of cellular glutathione for better adaptation to the oxidative stress [187]. Deletion of Nrf2 in a pancreatic cancer mouse model with mutant KRAS and p53 (KPC model) decreased formation of precancerous lesions and the development of pancreatic cancer, while increasing sensitivity to oxidative stress and chemotherapeutic agents [188]. Thus, the increased antioxidant system may potentially allow cancer cells to survive under oxidative stress that induces genomic instability, exacerbating cancer development. Recently, NRF2 was also reported to modulate mitochondrial function. Studies have shown that respiration rate and ATP production decreased in cells or mitochondria isolated from *Nrf2*-knockout mice but increased in their Keap1-knockout counterparts, identifying a critical role of NRF2 in cellular bioenergetics by controlling substrate availability for mitochondrial respiration [189]. NRF2 was also shown to be involved in mitophagy regulation and mitochondrial fatty acid oxidation. Overall, a growing body of evidence suggests that NRF2 can serve as a double-edged sword with both tumor-suppressive and tumor-promoting roles in cancer development [190,191,192].

## 6. Summary

Cancer cells exhibit an altered redox status and metabolism, which are associated with mitochondria as they are the major sites of ROS generation and energy metabolism. In this review, we have discussed mitochondrial dysfunction induced by the alterations of the mitochondrial genome, the TCA cycle enzymes, mitochondrial electron transport chain, and the associated oxidative stress. We have also discussed the aberrant oncogene and tumor suppressor signaling in the induction of mitochondrial dysfunction in cancers. Despite the prevalence of mtDNA mutations in different malignancies, their causal roles in cancer development remain incompletely understood and many questions still remain. It is imperative for us to gain a better understanding of why certain mutations appear more commonly in some cancers than in others. This may be in part due to the fact that different types of cancers have distinct underlying drivers and/or specific requirements in the path toward the malignant transformation. It has been known that oncogenic KRAS is the critical driver for the initiation and development of pancreatic cancers, while loss of tumor suppressors, such as PTEN and TP53, are major drivers of prostate cancer development. Mitochondrial dysfunction may be induced under either of these conditions but through distinct mechanisms. Certain control regions of mitochondrial single-nucleotide variants were recently found to co-occur with a gain of Myc oncogene in prostate cancer patients [86]. Different mtDNA mutations may impose divergent influences at varying degrees on the respiratory chain, with some mutations causing critical alterations of the respiratory chain and leading to transformation-promoting oxidative stress, while others may play lesser roles. The alterations of the electron transport chain affect not only electron transport, oxygen consumption, and ATP generation but also cellular redox status, metabolism, and apoptosis. It should be noted that a severe level of mitochondrial dysfunction may cause cell death and thus inhibit tumorigenesis, while mild mitochondrial dysfunction may enhance mitochondrial ROS generation and redox rebalance to stimulate cancer cell proliferation and invasiveness. Thus, a better understanding of the roles of mitochondrial dysfunction in cancer is essential for the future design of effective therapeutic strategies against diverse types of cancers.

## Figures and Tables

**Table 1:** Examples of mitochondrial enzyme dysfunction in cancer development.

Genes	Main Features and Mechanism	Cancer Types	References
IDH1/2 mutations	Accumulation of oncometabolites D-2HG, epigenetic alterations, and impaired mitochondrial respiration	Gliomas, glioblastomas, and AML	[11,12,13,14,15,16,17,18,19,20]
SDH mutations	Accumulation of succinate, inhibition of PHDs, and HIF1a stabilization	Pheochromocytoma, paraganglioma, and other cancers	[21,22,23,24,25,26,27,28,29,30,31,32,33,34]
FH mutations	Abnormal accumulation of fumarate, inhibition of PHDs, and HIF1a stabilization. Glutamine is used for heme synthesis. Glutamine is converted to aKG for citrate formation. Glutamine is also used to fuel TCA cycle and ATP production	Papillary renal cell cancer, leiomyosarcoma, pheochromocytoma, paragangliomas, and other cancers	[35,36,37,38,39,40,41,42,43,44,45,46]
SOD1^-/-^	Accumulation of O2-, abnormal mitochondria, oxidation of DNA, protein, and lipid, and decreased aconitase activity	Hepatocellular carcinoma	[47]
SOD2^+/-^	Mitochondrial oxidative damage, nuclear DNA/ mtDNA oxidation, and decreased mitochondrial membrane potential	Lymphoma, pituitary adenoma	[48]
